# Supplementary material for: Identification of the SHREK Family of Proteins as Broad-Spectrum Host Antiviral Factors
Source: Viruses. 2021 May 4;13(5):832. doi: 10.3390/v13050832 (PMC8147968; doi:10.3390/v13050832)
Supplement: Supplementary file 1 [file viruses-13-00832-s001.zip › viruses-1162070-supplementary.pdf]

viruses

MDPI

Article

Identification of the SHREK family of proteins as broad-spectrum host antiviral factors

Deemah Dabbagh [1], Sijia He [1], Brian Hetrick [1], Linda Chilin [1], Ali Andalibi [1] and Yuntao Wu [1,]*

Supplementary Material

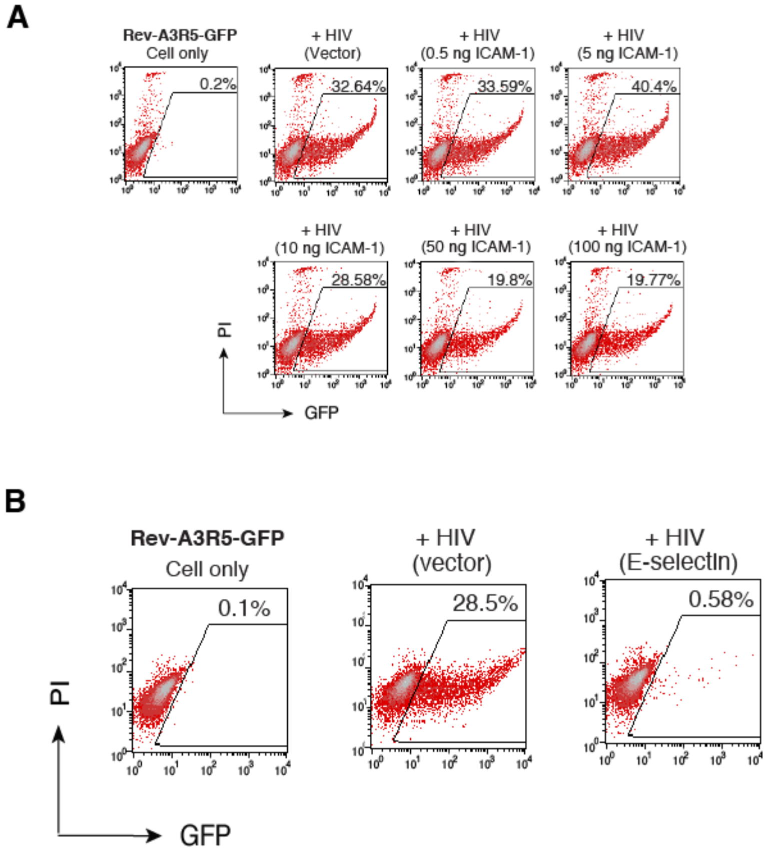

Figure S1. Effects of ICAM-1 and E-selectin on HIV-1 infectivity. (A) Effects of ICAM-1 on HIV-1 infectivity. HEK293T cells were co-transfected with 1 μg of HIV(NL4-3) DNA plus empty vector or the indicated doses of ICAM-1 vector DNA. Empty vector was used to maintain an equal DNA concentration. Virions were harvested at 48 h, normalized for p24 and 400 ng p24 inputs were used to infect Rev-A3R5-GFP cells. Infectivity was quantified by measuring the percentage of GFP+ cells by flow cytometry at 72 hours post infection. (B) E-selectin inactivates HIV-1 virion infectivity. HEK293T cells were cotransfected with HIV(NL4-3) DNA (1 μg) plus the vector expressing E-selectin or an empty vector (400 ng). Virions were harvested at 48 hours and normalized for p24, and viral infectivity was quantified by infecting Rev-A3R5-GFP indicator cells. Shown are the percentages of GFP+ cells at 72 hours post infection.

It has been shown that the infectivity of HIV-1 particles bearing ICAM-1 is greatly enhanced. This enhancement is through the interaction between ICAM-1 on the HIV-1 particle and LFA-1 on the target cells. We did not observe significant ICAM-1-mediated enhancement in the infection of Rev-A3R5-GFP cells, as shown in (A). It is possible that Rev-A3R5-GFP may not express high levels of active LFA-1, or there are other adhesion molecules over-expressed on Rev-A3R5-GFP that may overshadow the enhancement of ICAM-1/LFA-1.

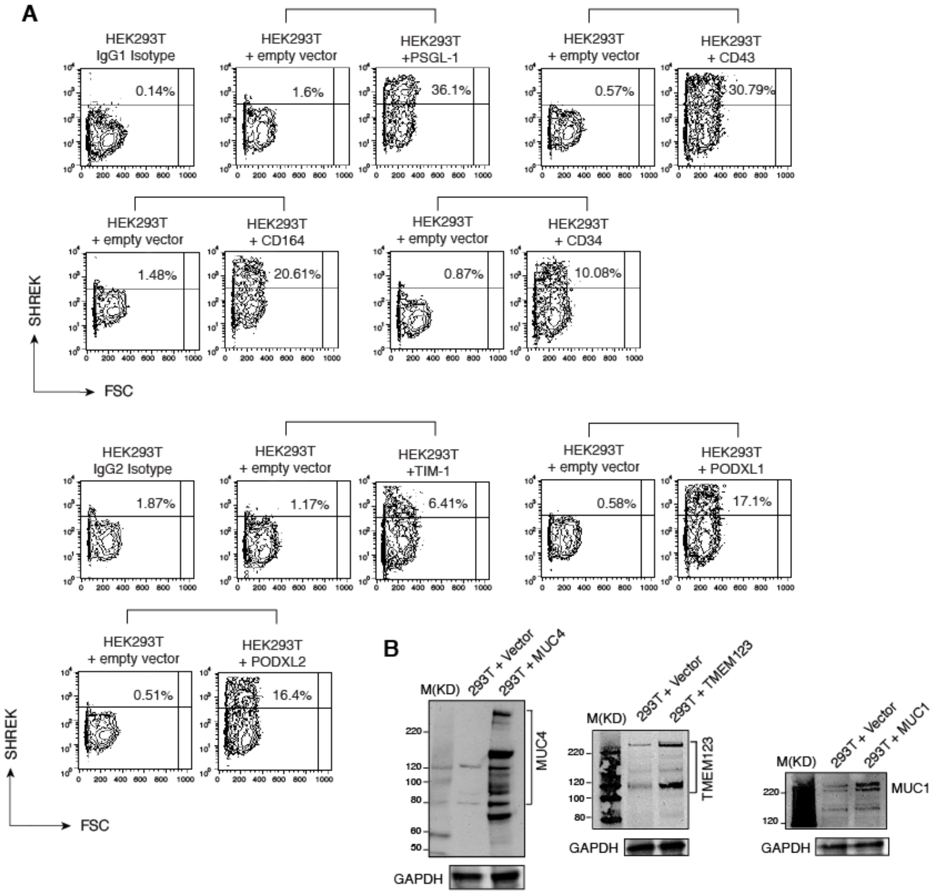

Figure 2. Expression of SHREK proteins in HEK293T cells. (A) HEK293T cells were transfected with 400 ng of either PSGL-1, CD43, CD164, CD34, TIM-1, PODXL1, or PODXL2. Expression of each protein was quantified at 48 hours by surface staining using antibodies against each of the indicated proteins. (B) HEK293T cells were transfected with 400 ng of MUC1, MUC4, TMEM123, or an empty vector. Expression of each protein was measured by Western blot using antibodies against MUC1, MUC4 or TMEM123.

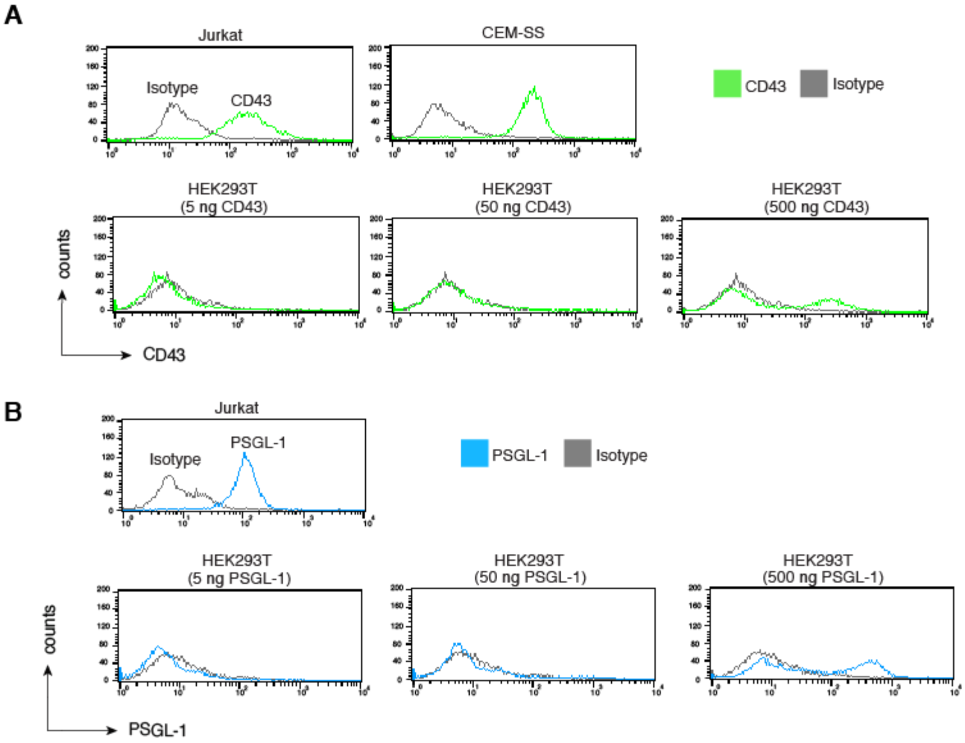

Figure 3. Comparison of CD43 and PSGL-1 expression levels in transfected HEK293T cells and Jurkat and CEM-SS T cells. HEK293T cells were transfected with the indicated doses of CD43 (A) or PSGL-1 DNA (B). At 48 hours post-transfection, cells were stained with either a PSGL-1 or CD43 primary antibody (1 million cells per staining), followed by staining with fluorescently labeled secondary antibodies, and then analyzed by flow cytometry. For comparison, Jurkat or CEM-SS cells were simultaneously stained and analyzed.

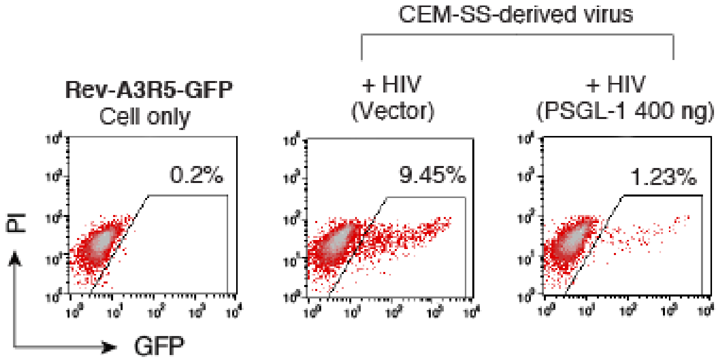

Figure S4. Over-expression of PSGL-1 in CEM-SS T cells leads to inactivation of virion infectivity. HIV-1 (NL4-3) particles were produced in CEM-SS T cells by co-electroporating of HIV-1 (NL4-3) DNA (1 µg) with an empty vector or PSGL-1 DNA (400 ng). Virions were harvested at 4 days post electroporation, and 9 ng p24 inputs of each virus were used to infect Rev-A3R5-GFP cells. Virion infectivity was quantified by measuring GFP+ cells by flow cytometry at 4 days post infection.

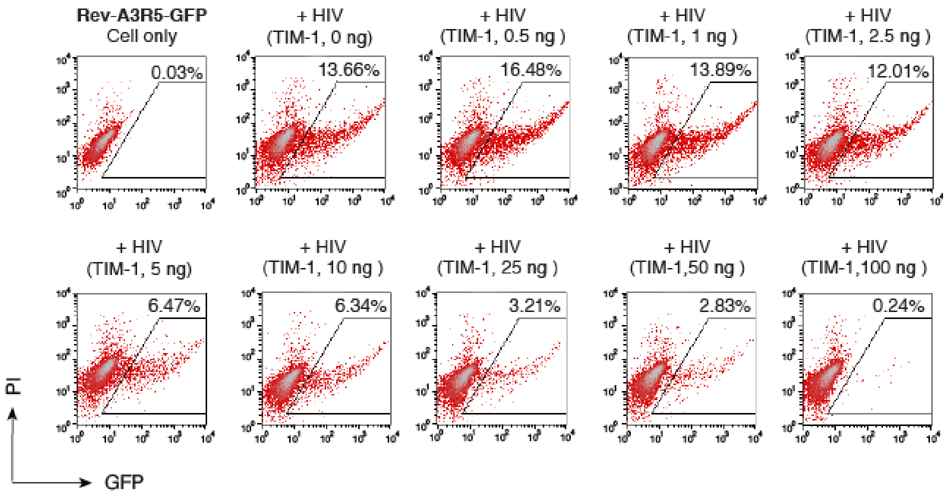

Figure 5. Dose-dependent inhibition of HIV-1 by TIM-1. HIV-1 particles were produced in HEK293T cells by co-transfection of HIV-1(NL4-3) DNA (1 µg) plus an empty vector or the indicated doses of TIM-1. P24-normalized virions were used to infect Rev-A3R5-GFP cells. The percentage of GFP+ cells was quantified by flow cytometry at 72 hours post infection.

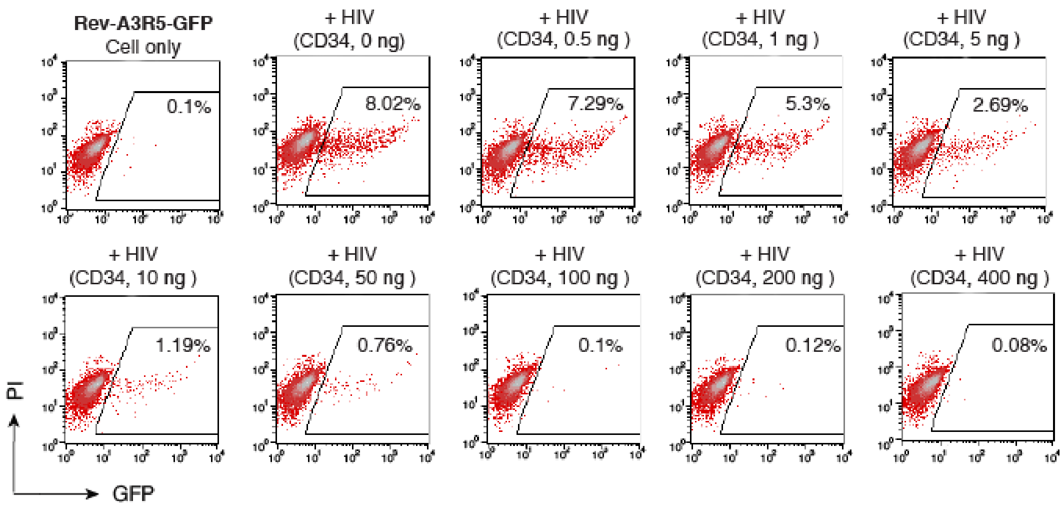

Figure S6. Dose-dependent inhibition of HIV-1 by CD34. HIV-1 particles were produced in HEK293T cells by co-transfection of HIV-1(NL4-3) DNA (1 µg) plus an empty vector or the indicated doses of CD34. P24-normalized virions were used to infect Rev-A3R5-GFP cells. The percentage of GFP+ cells was quantified by flow cytometry at 72 hours post infection.

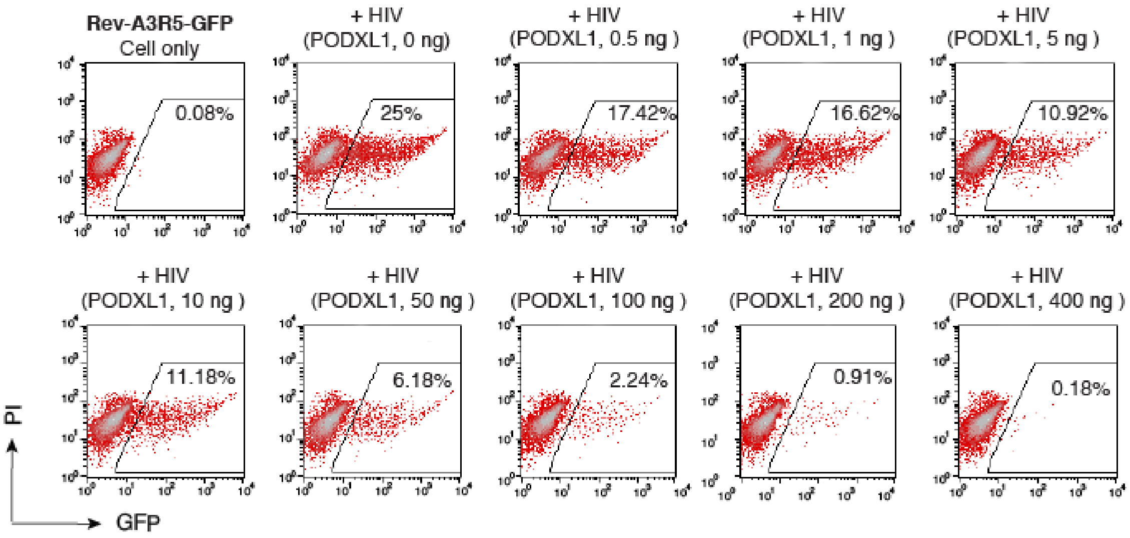

Figure S7. Dose-dependent inhibition of HIV-1 by PODXL1. HIV-1 particles were produced in HEK293T cells by co-transfection of HIV-1(NL4-3) DNA (1 µg) plus an empty vector or the indicated doses of PODXL1. P24-normalized virions were used to infect Rev-A3R5-GFP cells. The percentage of GFP+ cells was quantified by flow cytometry at 72 hours post infection.

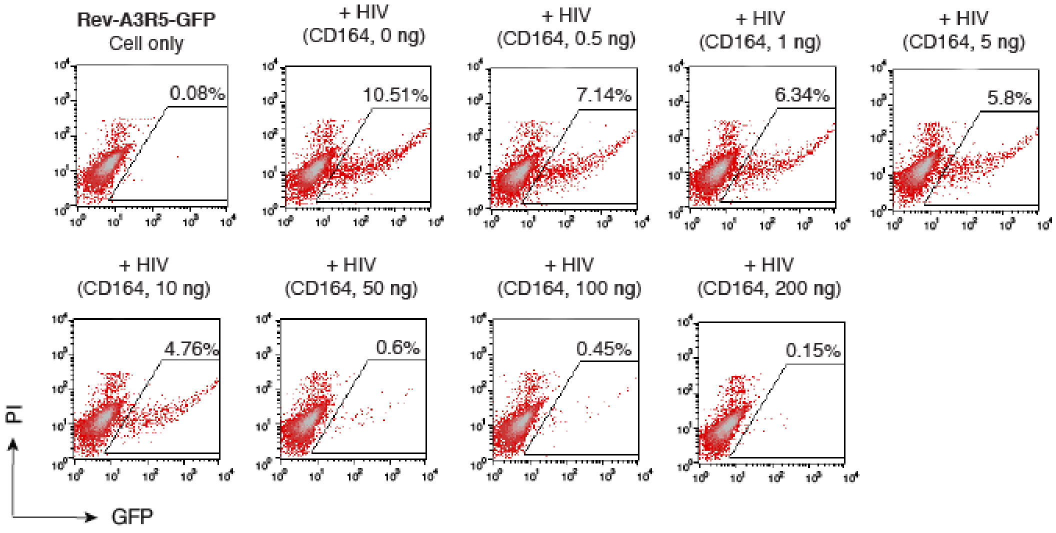

Figure 8. Dose-dependent inhibition of HIV-1 by CD164. HIV-1 particles were produced in HEK293T cells by co-transfection of HIV-1(NL4-3) DNA (1 μg) plus an empty vector or the indicated doses of CD164. P24-normalized virions were used to infect Rev-A3R5-GFP cells. The percentage of GFP+ cells was quantified by flow cytometry at 72 hours post infection.

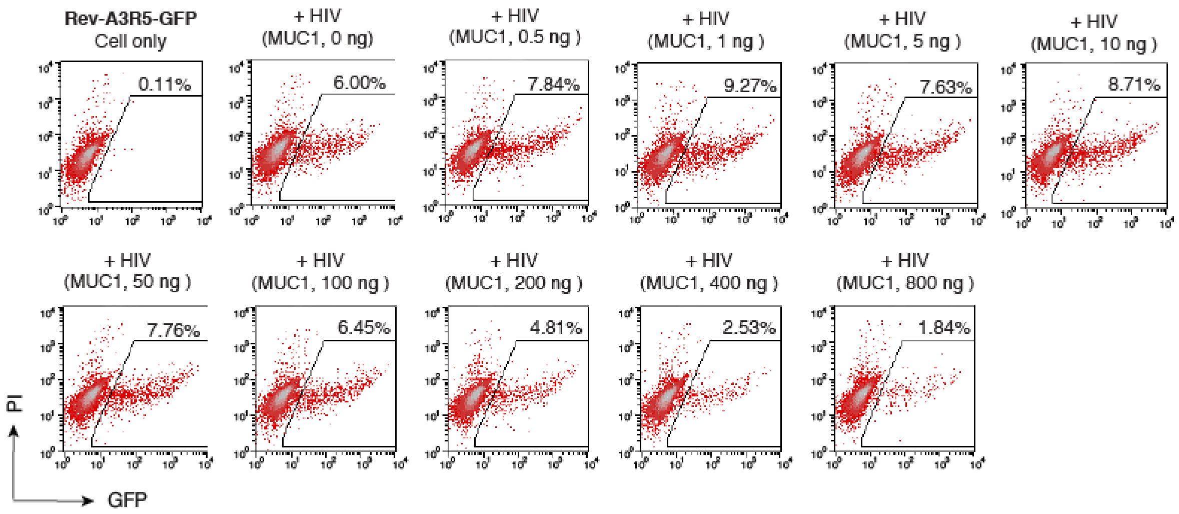

Figure S9. Dose-dependent inhibition of HIV-1 by MUC1. HIV-1 particles were produced in HEK293T cells by co-transfection of HIV-1(NL4-3) DNA (1 μg) plus an empty vector, or the indicated doses of MUC1. P24-normalized virions were used to infect Rev-A3R5-GFP cells. The percentage of GFP+ cells was quantified by flow cytometry at 72 hours post infection.

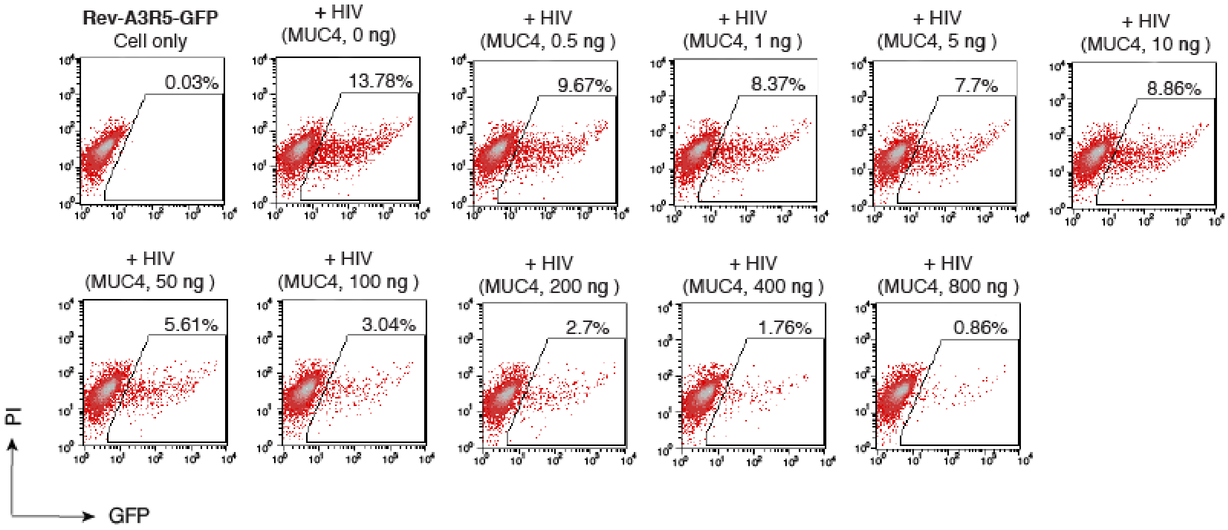

Figure S10. Dose-dependent inhibition of HIV-1 by MUC4. HIV-1 particles were produced in HEK293T cells by co-transfection of HIV-1(NL4-3) DNA (1 μg) plus an empty vector or the indicated doses of MUC4. P24-normalized virions were used to infect Rev-A3R5-GFP cells. The percentage of GFP+ cells was quantified by flow cytometry at 72 hours post infection.

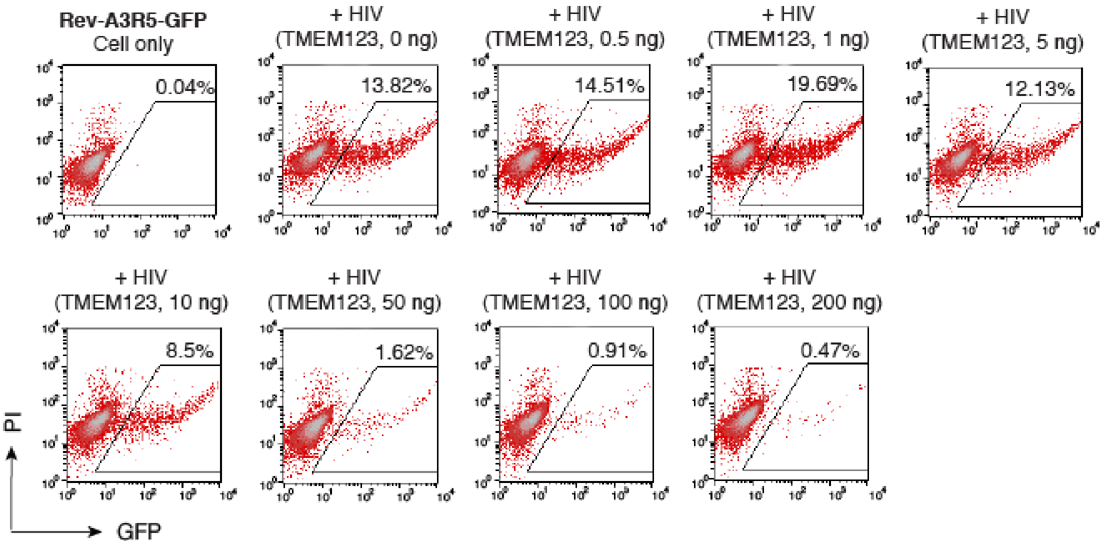

Figure S11. Dose-dependent inhibition of HIV-1 by TMEM123. HIV-1 particles were produced in HEK293T cells by co-transfection of HIV-1(NL4-3) DNA (1 μg) plus an empty vector, or the indicated doses of TMEM234. P24-normalized virions were used to infect Rev-A3R5-GFP cells. The percentage of GFP+ cells was quantified by flow cytometry at 72 hours post infection.

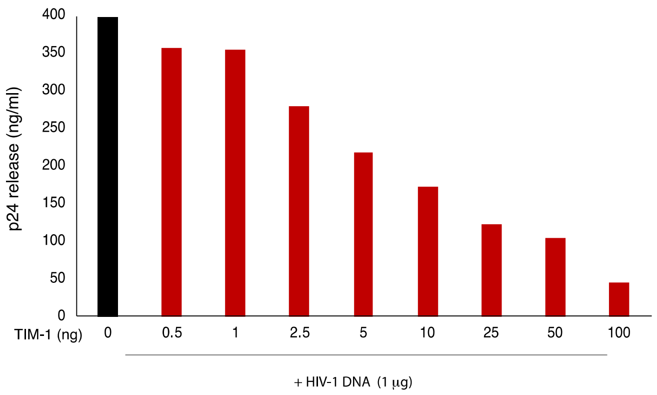

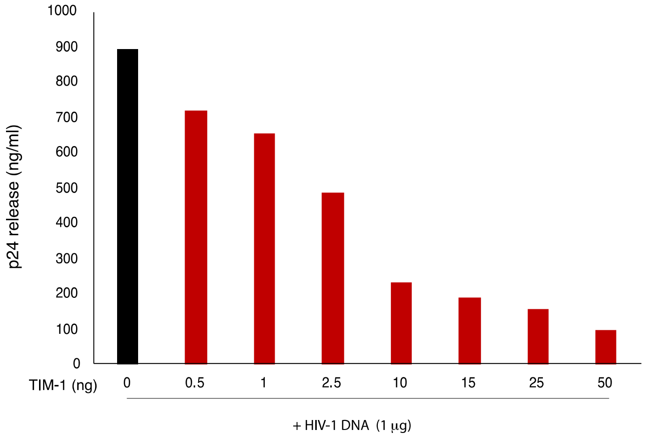

Figure S12. Effects of TIM-1 on HIV-1 release. HEK293T cells were co-transfected with HIV-1(NL4-3) DNA (1 µg) plus an empty vector or the TIM-1-expressing vector at the indicated doses. Viral supernatants were harvested at 48 hours post transfection and quantified for p24 levels by ELISA. Data from two independent transfections are shown.

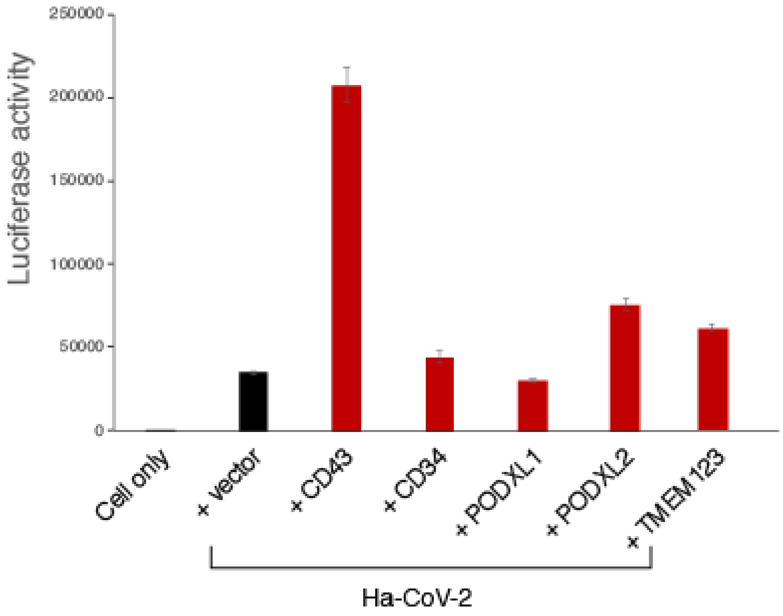

Figure S13. Lack of inhibition of Ha-CoV-2 virion infectivity by CD43, CD43, PODXL1, PODXL2, and TMEM123. Ha-CoV-2(Luc) particles were assembled in HEK293T cells by cotransfection of SARS-CoV-2 S-, M-, N- and E- expressing vectors (0.3 µg each), Ha-CoV-2(Luc) vector (1.2 µg), and each individual SHREK-expressing vector (CD43, CD34, PODXL1, PODXL2, or TMEM123) or an empty vector (1.6 µg). Virions were harvested, and used to infect

HEK293T(ACE2/TMPRSS2) target cells. Luciferase activity was quantified at 18 hours post infection. Data are presented as independent experiment triplicate means ± SD.

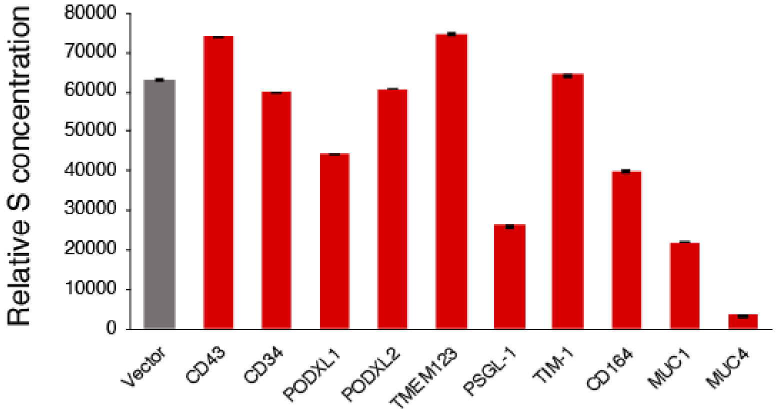

Figure S14. Effects of SHREK proteins on Ha-CoV-2 virion release and S protein incorporation. Ha-CoV-2(Luc) particles were assembled in HEK293T cells by co-transfection of SARS-CoV-2 S-, M-, N- and E- expressing vectors (0.3 µg each), Ha-CoV-2(Luc) vector (1.2 µg), and each individual SHREK-expressing vector or an empty vector (1.6 µg). Virions were harvested at 48 hours and levels of SARS-CoV-2 spike protein (S) were quantified by ELISA. Results are shown in ELISA triplicate means ± SD.
